# Isolated hepatitis B core antibody in HIV infected patients--can response to hepatitis B vaccine help to elucidate the cause?

**DOI:** 10.22088/cjim.9.4.328

**Published:** 2018

**Authors:** Mohammad Mahdi Majzoobi, Mojgan Mamani, Seyyed Hamid Hashemi, Hadis Gazan, Hamidreza Ghasemibasir, Mina Nikbakht, Farzaneh Esna-Ashari

**Affiliations:** 1 Department of Infectious Diseases, Hamadan University of Medical Sciences, Hamadan, Iran; 2 Brucellosis Research Center, Hamedan University of Medical Sciences, Hamadan, Iran; 3 Hamadan University of Medical Sciences, Hamadan, Iran; 4 Department of Pathology, Hamadan University of Medical Sciences, Hamadan, Iran; 5 HIV Clinic, Hamadan University of Medical Sciences, Hamadan, Iran; 6 Department of Community Medicine, Hamadan University of Medical Science, Hamadan, Iran

**Keywords:** HIV, Hepatitis B Virus, Isolated Anti Hepatitis B Core, Hepatitis B Vaccine

## Abstract

**Background:**

Concomitant hepatitis B and HIV infections are common. In some of these patients, HBcAb is the only serologic marker of hepatitis B. This study was conducted to elucidate the cause of isolated HBcAb in HIV-infected patients via hepatitis B vaccination.

**Methods:**

In this interventional study during 2014-15 in the HIV Clinic in Hamadan, thirty four patients with HIV infection and isolated HBcAb positive isolate, received hepatitis B vaccine and their responses to vaccination were investigated. Demographic data, stage of disease, and status of CD4 and HCV Ab were extracted from the patients' medical records and were entered in a checklist.

**Results:**

Of the 103 HIV positive patients, the prevalence of HBs Ag, and HBc Ab isolates were 6.79% (n=7) and 46.6% (n=48), respectively. All of the patients with isolated HBcAb were positive for HCV Ab. Among the 48 patients with isolated HBc Ab, 34 (70.8%) were available and examined for HBV DNA in serum samples. The result of PCR was negative in all. After the first round of hepatitis B vaccination, HBs Ab titer exceeded 10 International Units Per Liter (IU/L) in 58.8% of patients with isolated HBc Ab. With the completion of the three-dose of vaccine, this titer was observed in 97% of patients. Significant correlation was observed between titer of antibodies and values of CD4 cells.

**Conclusions:**

Due to favorable response to hepatitis B vaccination in HIV positive patients with isolated HBc Ab, false positive HBc Ab and recovery from previous infection were more probable than hidden hepatitis B.

With the increasing rate of HIV prevalence across many countries; this disease is considered globally pandemic. According to reports of the joint United Nations program on HIV/AIDS (UNAIDS), it is estimated that 35 million people worldwide were living with HIV virus by the end of 2013, of whom, about 95% live in the developing countries (1). Meanwhile, prevention, diagnosis and treatment of hepatitis B in patients with AIDS are particularly important. Recent studies have shown that the risk of mortality in patients with concomitant hepatitis B and AIDS infection is significantly higher than in patients with hepatitis B alone (2). All HIV patients should also be examined for hepatitis B infection. The evidence of active hepatitis B infection in AIDS patients exposes them to risk of cirrhosis, advanced liver diseases, and hepatocellular carcinoma. Treatment of hepatitis B in AIDS patients can be mainly aimed at preventing development of advanced liver diseases (2). A study showed that almost 80% of intravenous drug users with AIDS had serological markers of hepatitis B infection (3).

The presence of HBc Ab isolate in patients with AIDS indicates a hepatitis B serological change. HBc Ab isolate refers to the presence of antibody anti-core virus antigen in the absence of HBs Ab and HBs Ag (4). Some causes were suggested for isolated HBc Ab; first, isolated HBc Ab is seen in patients with chronic hepatitis B infection that its antigen level was too low to detect with serological examinations and both of HBs Ag and HBs Ab were negative (occult hepatitis B). Second, isolated HBc Ab was observed in patients who had recovered from hepatitis B or had prior vaccination but their HBs Ab level was too low for detection (5, 6) and third, it should be false positive due to the present cross-reacting antibodies in HIV, HCV or co-infection of them (7, 8). In patients whose HBs Ab level was too low to detect despite recovery from hepatitis B or prior vaccination, naturally, the antibody increased to higher than 10 IU/L after solitary vaccine dosage (9). 

In Chakvetadze et al.’ study on HIV patients with anti-HBc and CD4 count above 200, they reported that one month after hepatitis B vaccination, 22% of participants showed anti-HBs titer above 10 IU/L, and the rest were identified as primary responders. After the course of three vaccinations, about 60% of primary responders showed anti-HBs titer above 10 IU/L (10). There are still reports of unidentified issues in relation to the response rate of hepatitis vaccination in HIV patients. Accordingly, the present study aimed to investigate the different causes of isolated HBc Ab in HIV-infected patients by measurement of immune response to hepatitis B vaccination in the HIV clinic in Hamedan during 2015.

## Methods

The present interventional study was conducted on HIV- infected patients in the HIV clinic in Hamadan during 2014-2015. The study protocol was approved by the Ethics Committee of Hamadan University of Medical Sciences. First, the study participants underwent screening tests for hepatitis B. HBs Ag test was performed with a fourth generation ultra-version enzyme-linked immunoassay (ELISA) kit of DIA.PRO (Milano, Italy) using sample/control cutoff method (<0.9: negative, 0.9-1.1: equvocal, >1.1: positive). HBc Ab test was done with competitive enzyme immunoassay (ELISA) kit of DIA.PRO (Milano, Italy) using sample/control cutoff method (<0.9: negative, 0.9-1.1: equvocal, >1.1: positive). HBs Ab was measured using ELISA-kit of DIA.PRO (Milano, Italy) for quantitative determination of serum antibody level and the samples with a concentration higher than 10 mIU/ml considered as positive. CD4^+ ^lymphocytes count was detected by CyFlow Counter Sysmex Partec (Germany) device with flow cytometric and green laser excitation method using CD4/CD 4% easy count kit Sysmex reagents. 

Isolated HBcAb was defined as positive HBc Ab with negative HBs Ab and HBs Ag. Patients with isolated HBc Ab received HBV vaccination free of charge on three occasions: beginning of the study, one month and six months after the first vaccination. HBs Ab titer was measured three weeks after the first vaccination. In patients whose antibody titers increased higher than 10 IU/L after the first dose of hepatitis B vaccination, other doses of vaccine were not given. In patients whose antibody titer had not risen, second and third doses of HBV vaccine were injected and serum HBs Ab level was measured again three weeks after the third vaccination. A checklist was designed by researchers containing the date of referral, demographic details (age, gender), duration of infection, stage of disease, and status of CD4 and HCV Ab, which were extracted from the patients' records in HIV clinic and recorded in the checklist. 

Collected data were analyzed using SPSS-22. Quantitative data were described using mean and standard deviation while in qualitative data, frequency and percentage were used. For comparison of qualitative findings, we used chi-square test and McNemar’s test. With respect to quantitative findings, for antibody titer comparisons between the first and third vaccinations, we used Wilcoxon signed rank test; for antibody titer comparisons of HIV patients who did and did not receive antiretroviral treatment, we used Mann-Whitney U test as the antibody titer distribution did not turn out to be normal after checking for normality. We used Spearman correlation test to clarly the for relationship between antibody titer and the length of treatment, as well as CD4 and antibody titer after the first and third vaccinations. The significance level used in all tests was 5%.

## Results

**Figure 1:**
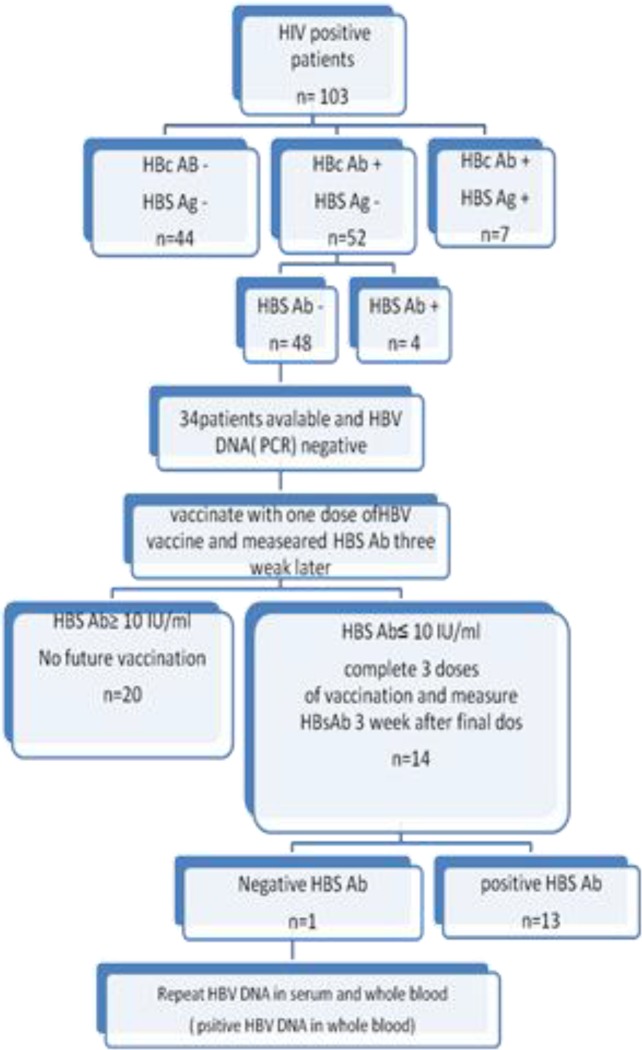
Flowchart of response to hepatitis B virus vaccine (HBV vaccine) in HIV positive patients with isolated hepatitis B core antibody (HBc Ab). HBs Ag = Hepatitis B surface Antigen, HBs Ab = antibody to hepatitis B surface antigen

In the present study of 103 HIV positive patients, 101 (98.1%) patients were men and two (1.9%) were women, with mean age 40.5±7.6 years. Participating patients were mostly in the 35-44 years age group (47.1%), where the 55-64 years age group contained the fewest patients (1%); the disease was not observed in the age group younger than 25 years old. All of 103 HIV patients in the HIV clinic were screened for hepatitis B. Of the 103 HIV infected patients, seven (6.79%) were HBs Ag and HBc Ab positive, in 44 (42.7%) patients HBs Ag and HBc Ab were negative, and finally 52 (50.5%) patients were HBs Ag negative and HBc Ab positive. The entire last group (i.e., negative HBs Ag and positive HBc Ab) had serologic evidence of hepatitis C infection and also history of hepatitis B vaccination before any complete screening for hepatitis B infection, where the mean time elapsed since vaccination was 6.26 years (1-10 years). Of the 52 patients in this group, only 4 (7.7%) were positive for HBs Ab and 48 (92.3%) were negative. Among the 48 patients that had serologic evidence of isolated HBc Ab, 34 (70.8%) were available and examined for HBV DNA; results of PCR procedure of serum samples of these 34 patients were negative (figure 1). 

With respect to the stage of HIV infection in these patients, 12 (35.3%) were in the first stage, 4 (11.8%) in the second, 12 (35.3%) in the third, and 2 (5.9%) in the fourth. The stage of the disease was unclear in 4 (11.8%) patients. Mean CD4 in patients with positive HIV and positive HBc Ab isolate was 337.12±203.4 (27-780).

HBs Ab titer was greater than 10 in (20 of 34) 58.82% of patients after the first vaccination. In those patients whose antibody titer did not increase after the first dose of vaccine, positive titer of HBs Ab was found in (13 of 14) 92.86% after the third vaccination round (p< 0.05) (table 1). 

**Table 1:** HBs Ab titer of patients with positive HBc Ab isolate after the first and the third hepatitis B vaccination

**HBs Ab titer after the first and the third hepatitis B vaccination**	**Value**	**Count**	**%**
HBs Ab 1	<10	14	41.18
	≥10	20	58.82
	Total	34	100
HBs Ab 3	<10	1	7.14
	≥10	13	92.86
	Total	14	100

Altogether, following vaccination HBs Ab titer in 33 (97%) patients reached above 10 IU/L, and remained negative in one patient (3%) after all three rounds of vaccination**.** For the noted patient, polymerase chain reaction for hepatitis B was repeated and was negative in serum but PCR of the whole blood sample was positive for hepatitis B infection (figure 1). 

The Spearman correlation test showed no significant correlation between HBs Ab titer in the first (r=-14, P=0.34) and the third (r=14, P=0.34) vaccinations and the duration of receiving treatment for AIDS. The mean and standard deviation of hepatitis B antibody titer was 90.95±141.05 after the first vaccination and 53.93±47.7 after the third. No significant difference was found between patients receiving and not receiving medication for AIDS in HBs Ab titer after the first or the third vaccination.

In this study, no significant difference was observed between CD4 (below or above 200) and antibody titer (below or above 10). 

The Spearman correlation test showed a significant correlation between CD4 and HBs Ab titers after the first and the third vaccination (p<0.05) (table 2).

**Table 2:** Distribution of CD4 and HBs Ab titer in patients with positive HBc Ab isolate after the first and third hepatitis B vaccination

**Vaccination round**	**Antibody titer**	**CD4**		**Total**	**Pvalue**
		**< 200**	**> 200**		
First	< 10	5	9	14	0.28*
	≥ 10	5	15	20	
	Total	10	24	34	
Third	< 10	0	1	1	0.09**
	≥10	5	8	13	
	Total	5	9	14	

* Chi-square test

** Fisher's exact test

## Discussion

The prevalence of isolated HBc Ab was 46.6% in this study, which is higher than values reported in other similar studies by Neau (17%) and Landrum (14%) (11, 12). It is estimated that isolated HBc Ab had been found in 10-20% of general population (13). Vaezjalali et al. reported that the prevalence of HBc Ab among healthy blood donors was 8% (14). In a study about patients with non-Hodgkin's lymphoma, Masarone reported that the prevalence of isolated HBc Ab positive was 26.9% (15); Sowole et al. reported that the prevalence of HBc Ab among dialysis patients was 20% (16). The prevalence of isolated HBc Ab among patients with HIV infection ranged from 17–81%, depending on the prevalence of HBV infection (8, 11, 17). Higher prevalence of isolated HBc Ab was seen in HCV carriers and IV drug users (3, 18). In the present study, all 34 patients with isolated HBc Ab were IV drug users and HCV carriers.

In different studies, higher prevalence of isolated HBc Ab in patients with HIV and HCV co-infection has been reported (8, 19-21). Some studies cite higher spread of occult hepatitis B infection (OBI) as the main reason for this observation (20, 21). They state that in case of concurrent HBV and HCV infection, replication of these viruses, and especially hepatitis B virus, are reduced that can cause OBI (21). On the other hand, some studies suggest the probability of false positive result and the cross effect of hepatitis C antibodies as the reason for higher spread of isolated HBc Ab in patients with HIV and HCV co-infection (8). The general findings of our study are more consistent with the latter hypothesis. 

In the present study, in HIV positive patients with positive HBc Ab isolate, 35.3% were in the first stage of the disease, 11.8% and 35.3% were in the second and third stages, respectively and 5.9% in the fourth stage. In 11.8% of patients, stage of disease was not mentioned in their records. In this study, mean CD4 count was found to be 337.12±203.4 cell/µL, which was less than that reported by Chakvetadze et al. (443 cell/µL), and that reported by Ramazani et al. (467.54±218.93 cell/µL) (10, 22). In a similar study, Jahanbakhsh et al. reported CD4 count of more than 500 cell/µL in 41.4% of patients, between 200 and 449 cell/µL in 52.1%, and less than 200 cell/µL in 6.5% (23). It seems that HIV infected patients in our region were found to be in the late stage; therefore, we need to improve screening system to detect HIV infected patients in the early phases of the disease. 

In the present study, hepatitis B antibody titer was greater than 10 in 58.82% of patients after the first vaccination; among the remaining, positive titer of HBsAb seen in 92.86% after the third vaccination round. Overall, following vaccination, HBsAb titer in 97% of patients reached above 10 IU/L, and remained negative in 3% of them after all three vaccinations. The increase in antibody titer was greater than that reported in studies by Masako Mizusawa (42%) (24) and Chakvetadze (60%) (10). 

The difference may be attributed to patient immunity to hepatitis B in the present study as all patients had history of hepatitis B vaccination. In contrast to the present study, in Jahanbakhsh et al.’s study, a significant correlation was found between immunity response to hepatitis B vaccination and CD4 count, so the patients with CD4 count greater than 500 showed better immunity response after hepatitis B vaccination (23). Similarly, in Mizusawa et al.’s study, a significant correlation was found between immunity response to hepatitis B vaccination and CD4 count at first vaccination (24). In Mizusawa’s study showed a rapid drop in HBs Ab in HIV positive patients 180 days after the third hepatitis B vaccination (24). 

In the present study, the mean of time-lapse since vaccination of the patients was 6.26 years. According to our results, HBs Ab titer after HB vaccination or recovery of previous infection may be dropped over time and then increased due to immune stimulation after revaccination. On the other hand, in occult hepatitis B, we do not expect an increase in HBs Ab titer in response to revaccination, hence, occult hepatitis B is improbable. One patient in our study did not have increase in his antibody titer after three hepatitis B vaccinations. Although the primary and secondary PCR study of his serum sample was negative for hepatitis B, PCR study of his whole blood sample was positive. 

In conclusion Given the satisfactory response of HIV positive patients with isolated HBc Ab to hepatitis B vaccination, false positive HBc Ab and recovery from previous infection were most probable than hidden hepatitis B. There is a need for further studies with more cases and compare isolated HBc Ab cases in HIV positive patients with and without hepatitis C infection for more comprehensive conclusions.
